# Prediction of Liner Metal Temperature of an Aeroengine Combustor with Multi-Physics Scale-Resolving CFD

**DOI:** 10.3390/e23070901

**Published:** 2021-07-15

**Authors:** Davide Bertini, Lorenzo Mazzei, Antonio Andreini

**Affiliations:** Department of Industrial Engineering, University of Florence, Via S. Marta 3, 50139 Florence, Italy; davide.bertini@unifi.it (D.B.); lorenzo.mazzei@unifi.it (L.M.)

**Keywords:** CFD, conjugate heat transfer, scale resolving, combustor, aeroengine, lean burn, metal temperature

## Abstract

Computational Fluid Dynamics is a fundamental tool to simulate the flow field and the multi-physics nature of the phenomena involved in gas turbine combustors, supporting their design since the very preliminary phases. Standard steady state RANS turbulence models provide a reasonable prediction, despite some well-known limitations in reproducing the turbulent mixing in highly unsteady flows. Their affordable cost is ideal in the preliminary design steps, whereas, in the detailed phase of the design process, turbulence scale-resolving methods (such as LES or similar approaches) can be preferred to significantly improve the accuracy. Despite that, in dealing with multi-physics and multi-scale problems, as for Conjugate Heat Transfer (CHT) in presence of radiation, transient approaches are not always affordable and appropriate numerical treatments are necessary to properly account for the huge range of characteristics scales in space and time that occur when turbulence is resolved and heat conduction is simulated contextually. The present work describes an innovative methodology to perform CHT simulations accounting for multi-physics and multi-scale problems. Such methodology, named U-THERM3D, is applied for the metal temperature prediction of an annular aeroengine lean burn combustor. The theoretical formulations of the tool are described, together with its numerical implementation in the commercial CFD code ANSYS Fluent. The proposed approach is based on a time de-synchronization of the involved time dependent physics permitting to significantly speed up the calculation with respect to fully coupled strategy, preserving at the same time the effect of unsteady heat transfer on the final time averaged predicted metal temperature. The results of some preliminary assessment tests of its consistency and accuracy are reported before showing its exploitation on the real combustor. The results are compared against steady-state calculations and experimental data obtained by full annular tests at real scale conditions. The work confirms the importance of high-fidelity CFD approaches for the aerothermal prediction of liner metal temperature.

## 1. Introduction

The mid-long range transportation is dominated by aviation, which is increasing year after year the number of passenger, thanks to the improvements in safety and the cost reduction of air travel. Recent ICAO forecasts [1], released before the extraordinary COVID-19 pandemic, estimated a growth of air traffic around 4.0% per year in the period 2020–2040. The public sensitivity to the environmental issues associated to aeronautics is deeply conditioning the aviation industry, which is facing the demand for lower emission aircraft. The most recent regulations established a −60% for NO${}_{x}$ versus the year 2000 baseline (ICAO-CAEP/6) and additional reduction for CO${}_{2}$ and nvPM (ICAO-CAEP/10). The goals of ACARE Vision 2020 have been revised with an horizon towards 2050 with the Flightpath 2050, with specific attention to NO${}_{x}$ and CO${}_{2}$.

In this context, the research efforts are devoted to improve the engine performance, mainly by increasing the Turbine Inlet Temperature (TIT) and the Overall Pressure Ratio (OPR). This trend greatly affects the hot gas path parts in the turbine and combustion chamber, which are subjected to stronger thermal stress that can undermine their integrity. As far as the combustor is concerned, the aforementioned development trend leads to higher coolant and flame temperature, reducing the cooling potential and increasing both convective and radiative heat loads. As a consequence, the thermal management of liners becomes more and more challenging.

The cooling system is strongly stressed, regardless of whether the combustor is based on a rich-burn or a lean-burn architecture. The requirements for controlling turbine inlet temperature profile, emissions, and metal temperature generate an intense competition for the management of the air flow available [2]. The residual flow split demands to control the liner temperature below the admissible limit of the material employed, as well as the extent of temperature gradients in the metal parts. In addition, the achievement of lower NO${}_{x}$ emissions is in contrast with the trends of TIT and OPR increase.

More and more aeroengine manufacturers are working to overcome the limitations of standard Rich-Quench-Lean (RQL) technology, which is approaching the limit as emissions reduction capability, limiting the chance to fulfill future standards for NO${}_{x}$, CO, and, most of all, particulate matter. The most promising solution seems represented by Lean Direct Injection (LDI) technology, in which most of the air coming from the compressor (i.e., more than 65% of the total inflow) is delivered to combustion primary zone through the fuel-air nozzle so to reach a homogeneous lean mixture and reduce temperature peaks [3].

In such conditions, the more effective cooling schemes are required for the thermal management of the liners. The open literature on combustor cooling reports a wide range of solutions, such as double-wall schemes, tiles, matrix cooling, and transpiration cooling [4]. During the past few years, multi-perforated liners (effusion cooling) have acquired more and more importance, thanks to the effective liner protection from hot gases through film coverage and heat removed within the holes due to the forced convection related to coolant delivery [5]. Additionally, it is characterized by lower weight and complexity compared to double-wall schemes, in addition to being less prone to dust clocking. This cooling technique has been widely investigated, both experimentally and numerically, on flat plate, standard mainstream conditions [5,6,7,8], as well as under the influence of swirling flows [9,10,11,12,13,14,15].

Considering the issues represented by cost and complexity of experimental campaigns at high temperature and pressure conditions, Computational Fluid Dynamics (CFD) has been adopted in the last few decades as reference tool to analyze the complex phenomena occurring in aeroengine combustors. Despite that, the complicated interactions among combustion, turbulence, radiation, and conduction heat transfer make the prediction of metal temperature a very challenging task, demanding for an exploitation of multi-physics method. RANS approaches for turbulence modeling, which represents the most common tool in the industrial framework for preliminary design steps, do not show the required fidelity to permit adequate accurate predictions. Indeed, turbulence has huge effects in swirling reacting flows on both chemistry, spray dynamics, and wall heat transfer. It is, therefore, recommended to adopt Scale Resolving models (SRS) to accurately address the aerothermal field in place of RANS formulations [16,17,18]. When dealing with Conjugate Heat Transfer (CHT) calculations, the use of the inherent unsteady Scale Resolving CFD models requires to manage the involved large interval of time scales. The longer characteristic time of solid heat conduction with respect to turbulent convection (*O*(1 s) against *O*(1 ms), respectively) makes fully-coupled methods infeasible in an unsteady framework. Therefore, to effectively handle this problem and reduce computational costs, a loosely coupling is preferred, where segregated simulation processes are adopted for each involved physics (i.e., flow field, radiation, and heat conduction) exchanging only the relevant quantities among domains at a given frequency. The open literature reports several approaches suitable for the analysis of long transients [19,20] or quasi-steady [21,22,23] metal temperature evolution.

The current works aims at reporting the development and benchmarking of the multi-physics tool named U-THERM3D, which allows the transient high-fidelity prediction of metal temperature in gas turbine combustors using the commercial package ANSYS Fluent adopting Scale-Resolving turbulence modeling. At this purpose, the test case considered is represented by the annular combustor designed by Avio Aero in the context of the European project LEMCOTEC, based on the lean burn injection system PERM (Partially Evaporated and Rapid Mixing). The full scale tests performed at the Central Institute of Aviation Motors (CIAM) provided fundamental validation results at different operating conditions in terms of outlet temperature maps, pollutant emissions, and wall temperature. Steady-state CFD analyses were carried out on the very same annular combustor with the THERM3D tool in Reference [24] to predict liner temperature, whereas Scale Resolving modeling was adopted in Reference [25] for an accurate description of aerothermal field focusing on profile temperature and pollutant emissions at the combustor outlet. The results revealed that CHT analyses can take advantage of high fidelity CFD models for turbulence. For this reason, the present work aims at demonstrating the feasibility of such high-fidelity approach for the detailed design of combustors.

The article is outlined as follows: The first part is focused to the description of the theoretical formulation behind U-THERM3D and its implementation in ANSYS Fluent. Then, the methodology is tested in terms of computational efficiency and verified on simplified test cases. Subsequently, the Avio Aero combustor is presented, along with the numerical modeling required and the most relevant results obtained.

## 2. Description of Proposed Methodology

Exploiting a past experience in the modeling of steady Conjugate Heat Transfer in the context of ANSYS CFX (i.e., THERM3D developed by Mazzei [26]), a new tool, called U-THERM3D, was developed to investigate multi-physics CHT problems in an unsteady fashion, taking advantage of the high-fidelity potential of Scale Resolving methods in predicting phenomena affected by turbulence. In order to have a wider library of models and more customization freedom, a migration from ANSYS CFX to ANSYS Fluent was required. In this context, an early application of THERM3D in ANSYS Fluent can be found in Reference [24], giving the basic framework for the development of the coupling code applied in the present work.

The founding concept of U-THERM3D procedure is a desynchronization of time advancements in the calculation of the involved phenomena, i.e., conduction within the solid, convection between fluid and solid, as well as radiation in the gas and its interaction with the solid. It worth emphasizing that the major benefits of SRSs lie in the convection phenomenon, which is driven by turbulence and can include several sub-phenomena, such as, for example, combustion and spray evolution, largely affected by mixing. Each involved physics is solved in separated processes which are coupled with a parallel strategy. Following Reference [22], at the prescribed coupling time step, a set of quantities are exchanged among domains in terms of instantaneous quantities. They consist of surface quantities for the solid-fluid and solid-radiation interactions, while volume quantities are necessary for the fluid-radiation coupling.

**Figure 1:**
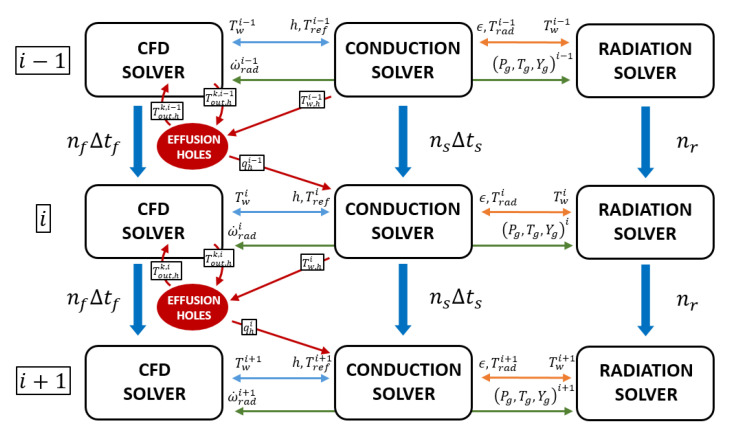
U-THERM3D parallel coupling strategy.

The user coding capabilities of ANSYS Fluent are used to customize the solver and develop the methodology. In particular, some User Defined Functions (UDFs) in C/C++ language were prepared to manage solvers synchronization and data exchange at interface patches, and this last step was also supported by dedicated scripts in the Ansys Fluent Scheme language. Indeed, as depicted in Figure 1, that shows the U-THERM3D procedure, although conduction and convection (CFD) simulations advance in time independently, the radiative heat transfer is handled with a steady approach, thanks to its relatively small characteristic time scale. The transfer of convective and radiative wall heat fluxes to the conduction solver requires a manipulation of the fluxes themselves. In particular, convective heat fluxes are expressed in terms of a convection boundary condition in the solid solver to obtain higher coupling stability [27]. The formulation is the following:

(1) $$q_{conv}^{\prime \prime }=h\left(T_{ref}-T_{w}\right),$$

where *h* is a coupling relaxation parameter, and $T_{ref}$ is a reference temperature with respect to wall heat flux computed in the fluid solver. Concerning radiation, a black-body model is employed, so that:

(2) $$q_{rad}^{\prime \prime }=\sigma_{0}\left(T_{rad}^{4}-T_{w}^{4}\right).$$

Consequently, the computation of radiative gas temperature $T_{rad}$ is required in order to set the radiative heat fluxes provided by the dedicated simulation. This approach is acceptable if the the solid is opaque, as in the present work. On the other hand, when the wall behaves as a semi-transparent medium, more accurate approaches can be adopted to improve the prediction of the radiative problem, as in Reference [28]. However, as demonstrated in Reference [29], when the Conjugate Heat Transfer is dominated by convection, the error neglecting wavelength dependency of radiative properties is minimum. As shown in Figure 1, return quantity from the solid is the wall temperature, used as a mixed Dirichlet-Robin BC by flow and radiative field computations. Even though this condition does not ensure energy conservation at the interface, it provides a stable coupling. Again, more advanced approaches can be found in literature. One of these is illustrated in Reference [23] and is able to respect energy balance imposing Dirichlet BCs both in the solid and in the convection solver. In the present strategy, however, using a high coupling frequency the error is definitely below the global one given by the methodology itself. Field variables from the CFD solver, i.e., composition, temperature, and gas pressure, are sent to the radiative solver which, as a result, provides for the flow field the energy source due to emission and absorption.

Even if, in the present work, the above described methodology is applied for the multi-physics investigation of a lean-burn combustor, the basic architecture of the tool, being developed as a framework where different solvers can be coupled, could be potentially used for other applications modifying few code lines. Indeed, compared against the available capabilities of ANSYS fluent, U-THERM3D is proposed as a general customizable framework to include your own physics solved in a dedicated instance. For example, in the developed approach for CHT problems on combustor liners, the additional modelings that actually are not possible with the standard solver are the decoupled solution of radiative problem using a mesh that is coarser than fluid domain one. To enforce this last sentence, Figure 1 highlights also the integration in the workflow of a dedicated solver for the heat sink effect of effusion holes that was a key task of the steady state methodology THERM3D [26]. It is based on the computation of the overall energy balance across the hole using a correlative approach. The boundary conditions at inlet and outlet patches are evaluated through a dedicated imprinting technique, where the coolant is injected with a specified direction and velocity. As it will be discussed in the following, this feature is not adopted in the current investigation: the interested reader is referred to References [24,26] for further details. An example of application of a similar methodology can be found in Reference [30] for turbine blade cooling.

### 2.1. Efficiency in High-Performance Parallel Computing

The present approach shows the best performances in massively parallel computing. For this reason it is suitable in the prediction of the complex thermal interactions within gas turbine combustors. Nonetheless, a good balancing in terms of CPU is required between the involved solvers to minimize the queue time and, therefore, the waste of computational resources. This goal is pursued with a synchronization in CPU time in place of physical time.

Given a certain number of processors *P*, a fully coupled approach uses all the available resources to solve the complete set of equations. On the other hand, in a loose parallel coupling, a portion of the total processors is devoted to solve only the equations modeling a certain phenomenon. In a convective-conduction problem, for instance, hypothesizing a perfect scaling for both the solvers, load balancing requires that the processors $P_{f}$ and $P_{s}$ assigned to fluid and solid computations, respectively, must be such that [30]:

(3) $$\frac{P_{f}}{P_{f}+P_{s}}=\frac{P_{f}}{P}=\frac{1}{1+\frac{T_{s}}{T_{f}}}.$$

Here, $T_{f}$ and $T_{s}$ are the execution times of the fluid and solid solvers on one processor to compute physical times $\Delta \tau_{f}$ and $\Delta \tau_{s}$, respectively.

A deeper analysis to quantify the advantages in terms of saved computational resources can be performed for the present methodology, keeping the hypothesis of ideal scaling. In the following, $I_{x}$ is the execution time of one time step of the “*x*” solver on a single grid element exploiting a single processor, whereas $N_{el,x}$ is the corresponding number of elements. Given a CHT problem involving convection (*f*), radiation (*r*), and conduction (*s*), a strongly coupled approach would require an execution time $\Delta T_{cht}$ estimated as:

(4) $$\Delta T_{cht}=\frac{\Delta \tau_{max}}{\Delta t_{min}}\frac{N_{el,f}\left(I_{f}+n_{r,cht}I_{r}\right)+N_{el,s}I_{s}}{P},$$

where $n_{r,cht}$ represents the number of iterations in a fluid time step for the radiative computation, and it is introduced because radiation is solved in a steady fashion, unlike the other solvers. The maximum physical time $\Delta \tau_{max}=max\left(\Delta \tau_{f},\Delta \tau_{r},\Delta \tau_{s}\right)$ is driven by the solid. The minimum time step $\Delta t_{min}=min\left(\Delta t_{f},\Delta t_{s}\right)$, instead, is limited by the fluid solver.

Moving to the U-THERM3D approach, the parallel scheme provides a different form of the execution time. Defining the solution time of the different solvers as follows:

(5) $$\Delta T_{f}=\frac{\Delta \tau_{f}}{\Delta t_{f}}\frac{N_{el,f}I_{f}}{P_{f}},\Delta T_{r}=\frac{\Delta \tau_{f}}{\Delta t_{f}}n_{r,uth}\frac{N_{el,r}I_{r}}{P_{r}},\Delta T_{s}=\frac{\Delta \tau_{s}}{\Delta t_{s}}\frac{N_{el,s}I_{s}}{P_{s}},$$

an expression for the execution time $\Delta T_{uth}$ can be derived:

(6) $$\Delta T_{uth}=max\left(\Delta T_{f},\Delta T_{r},\Delta T_{s}\right)+n_{cpl}T_{com},$$

where $n_{r,uht}$ in Equation (5) represents the total number of iterations performed by the radiative solver during a fluid time step, and it is commonly set less than 1. The second right-hand term in Equation (6) is added to account for a non-ideal communication method, that depends on the number of coupling $n_{cpl}$, as well as the execution time $T_{com}$ for the communication on a single coupling. The characterization of $T_{com}$ is not trivial because it can depend on the size of the exchanged data, the communication scheme, and, last but not least, the computational power estimated as the number of processors, the CPU quality, and the communication protocol. For a perfect synchronization in CPU time, queue time of the solvers should be avoided, and then all the simulations stop in the same instant and restart immediately. From a mathematical point of view, this condition is represented by:

(7) $$\Delta T_{f}=\Delta T_{r}=\Delta T_{s}.$$

To have a gain in the use of U-THERM3D approach, $\Delta T_{uth}$ must be such that:

(8) $$\Delta T_{uth}<\Delta T_{cht}.$$

**Figure 2:**
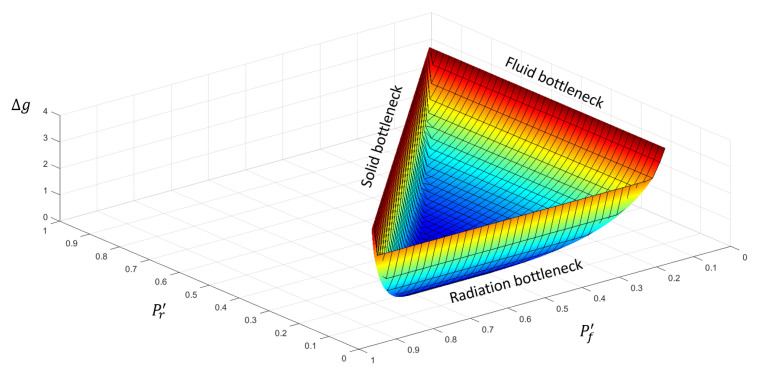
Relative computational losses for U-THERM3D in the case of non-ideal balancing as function of $P_{r}^{\prime }$ and $P_{f}^{\prime }$ for typical value of the quantities in Equation (5) for a combustor.

A lack of attention in the distribution of the processors between solvers has a negative effect on the performance of the present method. Hence, the degree of performance losses Δg can be defined as:

(9) $$\Delta g=\frac{\Delta T_{uth}-\Delta T_{uth}^{opt}}{\Delta T_{uth}^{opt}},$$

where $\Delta T_{uth}^{opt}$ is the minimum execution time provided by a perfect load balancing. Figure 2 shows the trend of Δg as function of the load balance (i.e., the fraction $P_{x}^{\prime }=P_{x}/P$ of *P* demanded to the “*x*” solver, or $P_{f}^{\prime }$ and $P_{r}^{\prime }$) for typical parameters of a combustor. Obviously, only values of $P_{f}^{\prime }$ and $P_{r}^{\prime }$ such that $P_{f}^{\prime }+P_{r}^{\prime }<1$ are permitted. The ideal load balancing for the hypothesized parameters corresponds to $P_{f}^{\prime }=0.68$, $P_{r}^{\prime }=0.2$, and $P_{s}^{\prime }=0.12$, but the computational time can increase beyond the 400% when different values are chosen. The location of the optimal point is critical because a small change in $P_{r}^{\prime }$, as well as a small increase of $P_{f}^{\prime }$, can lead to abrupt worsening of performance. This occurs because the execution time becomes driven by solid or radiation solvers depending on the value of $P_{r}^{\prime }$. The three bottlenecks are evident in Figure 2: the rate of performance losses when more processors are devoted to a solver is inversely proportional to the cost of the simulation. Indeed, while solid and radiation show steep increase of Δg, fluid solver is less sensible to a reduction in the number of processors. Moreover, for lower values of $P_{f}^{\prime }$, a wider margin of $P_{r}^{\prime }$ is allowed without a further loss of performance.

It is worth remembering that the present analysis was performed with the main hypothesis of ideal communication between the solvers, corresponding to $T_{com}=0$ in Equation (6). However, especially when a high coupling frequency is required, this parameter can strongly affect the computational performance of a loose coupling approach. For this reason, one of the main goals in the development of such a tool is the minimization of inter-communication time through advanced methods exploiting, for instance, MPI protocol. However, optimization of these aspects are not the focus of the present work and in U-THERM3D data are exchanged using standard tools provided by ANSYS Fluent, such as interpolation and profile files. Further effort is required from this perspective.

### 2.2. Tool Verification

**Figure 3:**
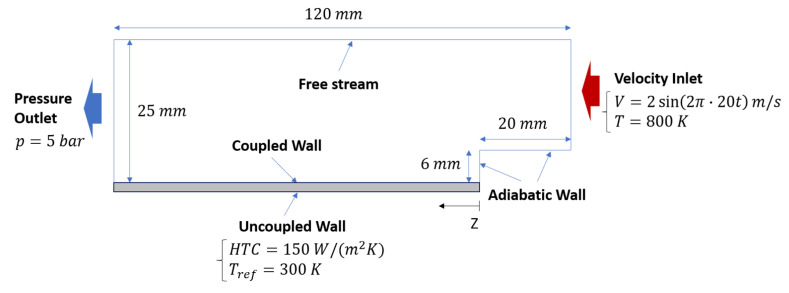
Geometric details and boundary conditions of the backward-facing step problem.

An assessment was performed on the backward-facing step, depicted in Figure 3, exploiting a URANS k−ϵ framework to limit the computational effort. A 2-D conjugate heat transfer problem between air and a solid was investigated limiting the domain size in the spanwise direction and applying symmetry conditions. The set of boundary conditions was completed by a constant temperature and a sinusoidal velocity at the inlet, whereas a constant pressure was applied at the outlet. The 1.5 mm-thick solid was coupled with the fluid domain on one side, while a convective boundary condition was provided on the other side. As a result, the location of the stagnation point is time-dependent with a consequent unsteady wall heat flux and temperature. The mean wall temperature predicted by U-THERM3D was compared against the results provided by a strongly coupled approach. To have an affordable computational cost of the reference simulation, material properties of the solid were chosen to reduce its characteristic time scale and are summarized in Table 1. The two domains were coupled every 10 time-steps of the fluid (0.01 s) solver that corresponds to a time advancement of 0.5 s for the solid. Therefore, the resulting acceleration factor is 50 in the present loosely-coupled simulation. The initial solid temperature was set according to the steady RANS results to avoid the thermal transient. Hexahedral meshes of 800 K and 20 K elements was built to solve the fluid and solid, respectively. To model wall heat fluxes and shear stresses, scalable wall functions were adopted. Independently, by the accuracy of the numerical setup, the main aim in this phase is the assessment of U-THERM3D as a valid alternative of a standard strongly coupled CHT approach. Hence, by using the same mesh and numerical setup, as well as boundary conditions, in both the unsteady simulations, the effect of a different coupling on the prediction of wall temperature can be isolated.

**Figure 4:**
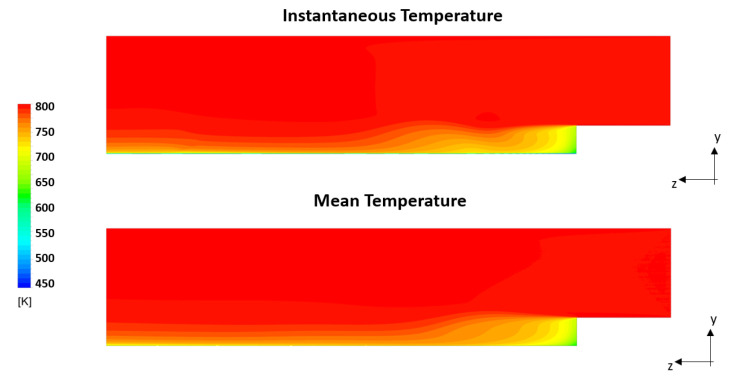
Instantaneous and mean temperature resulting form the U-THERM3D simulation for the backward-facing step problem.

**Figure 5:**
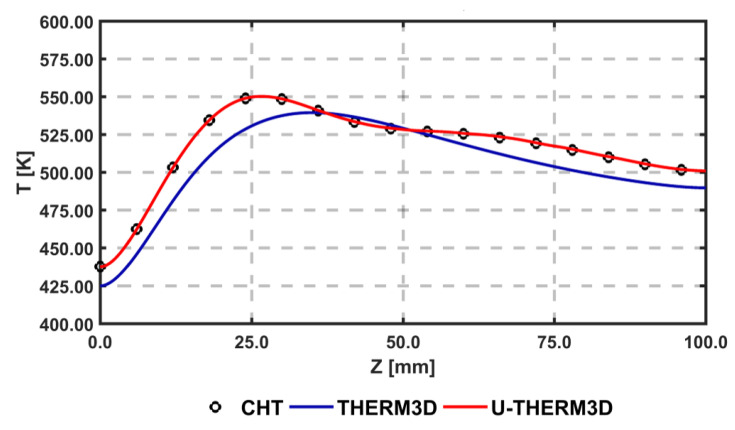
Axial distribution of temperature at the coupled wall obtained with a strongly coupled method (CHT), U-THERM3D, and THERM3D.

Figure 4 shows on the axial plane the cooling of gas due to the heat transfer at the wall for the U-THERM3D simulation. The different distribution of instantaneous and mean gas temperature are related to the fluctuating inlet velocity that modifies over time the size of the recirculation zone. The comparison of axial distribution of temperature at the coupled wall between the two approaches is reported in Figure 5. In addition, the strongly coupled (CHT) and the U-THERM3D simulations are superimposed on the result of THERM3D to highlight the limits of a steady loosely coupled approach. The peak wall temperature, located around the stagnation point where the heat transfer coefficient is maximum, moves upstream in unsteady simulations. The main interesting result, however, is the perfect matching of the present method with a more computational expensive one as the strongly coupled simulation, demonstrating the consistency in terms of energy balance.

## 3. Experimental Test Case

### 3.1. The LEMCOTEC Combustor

**Figure 6:**
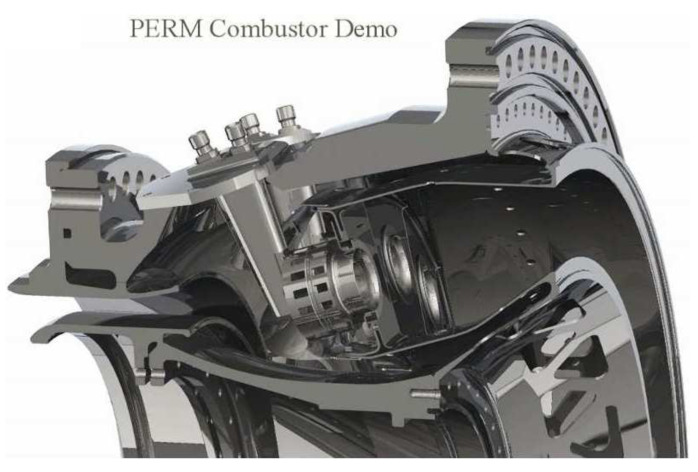
Avio Aero’s NEWAC combustor.

The case study investigated in this work is a straight-through annular combustor developed and tested in the research project LEMCOTEC [31]. The combustor layout is based on a dump diffuser delivering air to the cowl and inner and outer annuli. Air passing through annuli is injected in the chamber through a regular staggered pattern of inclined effusion holes, with a limited quota directed to bleeding ports in the final part of each annulus. The air admitted to the cowl feeds the burner and the dome cooling system based on jets impinging and heat shield. The combustor is based on a lean burn concept and it could be seen as an evolution of the unit developed in an earlier EU research program named NEWAC; a sketch of that chamber is reported in Figure 6. The new combustor is based on an optimized shape of the combustion volume linked to an innovative injection system and a revised air flow split. In addition, the liners’ cooling system was revised by adopting an improved effusion cooling scheme capable to reduce the amount of cooling air, with a reduced impact on unburned pollutant species, without affecting hardware durability.

**Figure 7:**
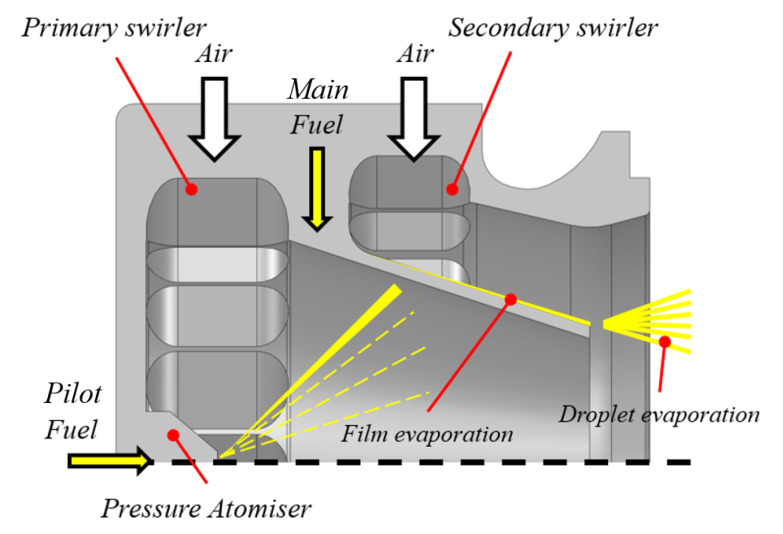
PERM injection system adopted on the LEMCOTEC combustor.

The LEMCOTEC combustor is based on an overall lean, swirl-stabilized, spray flame. The flame is realized by lean direct injection burner called PERM (Partial Evaporation and Rapid Mixing). The PERM concept is designed to work at intermediate OPR (between 20 and 35) and is based on two co-rotating radial swirlers encompassing a pre-filming atomizer (representing the main fuel injection device) and a pressure atomizer located at nozzle axis, which is adopted for main flame piloting to improve stability at low power conditions (see Figure 7). A variable pilot-to-main fuel split is planned during engine mission with pilot fuel percentage below 15% at max Take-Off conditions [32].

A complete full annular test campaign was carried out at the CIAM center (Central Institute of Aviation Motors) to validate combustor up to full load conditions. In this numerical study, the Approach and max Take-Off conditions are considered for modeling validation; in Table 2, the most significant operating conditions for the analyzed test points are listed.

### 3.2. Available Measured Data

**Figure 8:**
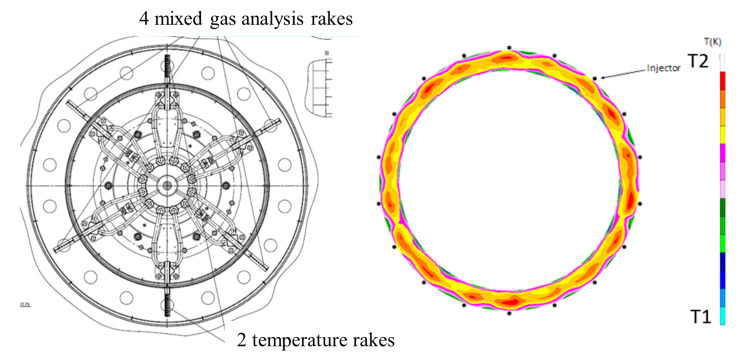
Scheme of the rotating probes installed at combustor outlet (**left**) and an example of measured temperature distribution for the Approach condition (**right**).

The metal temperatures of combustor liners were measured by several thermocouples located on the cold side of both inner and outer liners. The thermocouples were located on different sectors varying angular position: for the sake of simplicity during comparison with numerical results, thermocouples were grouped and referred to the same ideal sector of the combustor. The temperature pattern at combustor outlet and the exhaust emissions were measured, thanks to a rotating probe located at combustor exit (see Figure 8). The probe was also used to collect exhaust gas emissions which are not analyzed in this study; a detailed discussion and numerical prediction of combustor emissions are subject of a previous work from the authors, also based on the use of Scale-Adaptive Simulation (SAS) [25].

## 4. Numerical Modeling

All the simulations discussed in this work were carried out with the commercial package ANSYS Fluent version 17.1. In the following sections, detailed descriptions of the adopted numerical models are reported.

### 4.1. Turbulence Modeling

The Scale Adaptive Simulation approach was here considered to describe the turbulent nature of the flow. In particular, the SAS-SST model is here considered. The method can be seen as an advanced Unsteady Reynolds Averaged Navier-Stokes (URANS) formulation, where the capability to solve turbulent scales is obtained by locally reducing eddy viscosity, thanks to a source term $Q_{SAS}$ in the ω-equation conditioned by the von Karman length scale $L_{vK}$ [33]:

(10) $$Q_{SAS}=max\left[\rho \zeta_{2}\kappa S^{2}{\left(\frac{L}{L_{vK}}\right)}^{2}-C\frac{2\rho k}{\sigma_{\phi }}max\left(\frac{\left|\nabla \omega \right|}{\omega^{2}},\frac{\left|\nabla k\right|}{k^{2}}\right),0\right],$$

with $\zeta_{2}$, $\sigma_{\phi }$, and *C* being model constants, κ is the von Karman constant, and *S* is the strain rate tensor. The quantity *L* in Equation (10) is the integral turbulent length scale which is computed, as in a RANS calculation, starting from the the modeled turbulence. $L_{vK}$ is defined as:

(11) $$L_{vK}=\kappa \left|\frac{U^{\prime }}{U^{\prime \prime }}\right|$$

and depends on the first and second derivatives of the velocity field through:

(12) $$|U^{\prime }|=\sqrt{\frac{\partial U_{i}}{\partial x_{j}}\frac{\partial U_{i}}{\partial x_{j}}};\;\left|U^{\prime \prime }\right|=\sqrt{\frac{\partial^{2}U_{i}}{\partial x_{j}^{2}}\frac{\partial^{2}U_{i}}{\partial x_{k}^{2}}}.$$

Scale Adaptive Simulation is one of the most effective hybrid scale resolving formulation, as it permits to preserve the standard URANS approach where the flow field is less susceptible to instability, but it is able to behave as a LES-like solution in the case of highly unsteady flows, such as jets in cross flow and swirling flows. This characteristic is particularly effective in the case of wall bounded flows, allowing to limit computational cost, thanks to reduced mesh requirements in the near wall regions, where a RANS approach is retained. The SAS method also offers some advantages with respect to other hybrid RANS-LES strategies, such as the Detached Eddy Simulation (DES), with a lower sensitivity to mesh size and the absence of grid-induced separation phenomena. The SAS model controls the turbulence length scale according to the local flow inhomogeneities by the means of the scale $L_{vK}$. Nevertheless, differently from LES, the SAS approach is not based on filtered equations, so the grid size is not directly responsible for scale resolution, being that the dynamically adjusted quantity $L_{vK}$ is capable to reproduce the turbulence spectrum. However, it is obvious that proper temporal and spatial discretizations need to be assured to properly describe smallest scales in the regions where LES-like solution is requested; this will avoid nonphysical damping of the energy dissipation process with an overpredicted eddy viscosity preventing to catch the actual unsteadiness of the flow.

### 4.2. Turbulent Combustion Modeling

Following a series of previous successful validation studies carried out by the authors in the recent past (see References [17,18]), the Flamelet Generated Manifold (FGM) combustion model was considered to describe the turbulent flame occurring in the combustor. The FGM model is a flamelet-based approach where an external look-up table is generated by solving a set of laminar one-dimensional flamelets. The manifold is created by considering two fundamental parameters, i.e., the mixture fraction *Z* and the progress variable $c=Y_{c}/Y_{c,eq}$, where $Y_{c}=Y_{CO}+Y_{CO_{2}}$ is the un-normalized variable used to track the evolution from unburnt to burnt states. The generic two-dimensional manifolds ϕ(Z,c) were realized using non-premixed flamelets in opposed jets configuration: the reactivity of the flamelet was explored by varying the flame stretch from equilibrium up to flamelet extinction. The flamelets were solved assuming adiabatic conditions but non-adiabatic effects in the turbulent flame are modeled according to a enthalpy defect approach [34]. To account for the turbulence chemistry interaction, a joint-PDF approach was used by assuming independent double β-Probability Density Functions (β-PDF) for both mixture fraction and progress variable [35]. For the generic manifold quantity ψ(c,Z), the ensemble averaged value is obtained by the following integration:

(13) $$\begin{array}{c} \tilde{\psi }=\int \int \;\psi \left(c,Z\right)PDF\left(c,\tilde{c},\tilde{c^{\prime \prime }{}^{2}}\right)PDF\left(Z,\tilde{Z},\tilde{Z^{\prime \prime }{}^{2}}\right)\;dcdZ, \end{array}$$

where $\tilde{c}$, $\tilde{Z}$ and $\tilde{c^{\prime \prime }{}^{2}}$, $\tilde{Z^{\prime \prime }{}^{2}}$ are, respectively, the mean values and variances of progress variable and mixture fraction: these four variables are obtained by solving dedicated transport equations. Mixture fraction equation includes a source term to account for fuel vapor produced by spray evaporation, while the progress variable source term is directly obtained by the finite rate sources of the chemical species used for the definition of the progress variable and according to the integration described in Equation (13). The Jet A-1 fuel used for the experimental tests of the investigated LEMCOTEC combustor was here modeled as pure n-decane ($C_{10}H_{22}$), for which a detailed kinetic mechanism based on 96 chemical species and 856 elementary reactions was used [36].

### 4.3. Spray Modeling

A standard Lagrangian-Eulerian approach was considered to track the evolution of sprays droplets injected just downstream of the primary break-up region occurring at the pre-filmer tip. Injected droplets are tracked accounting for the two-way coupling with the continuous phase in terms of momentum, heat transfer, phase transition, and secondary atomization. The secondary break-up of the droplets, occurring in the shear layer regions produced by the high speed annular jet of the swirler, is here faced by using the WAVE model [37], suitable for the high Weber number conditions observed (i.e., We > 100). No specific models have been activated to account for turbulent dispersion being the Scale Adaptive Simulation able to directly solve most of the energy-carrying vortices. The widely accepted model proposed by Abramzon and Sirignano [38] was considered to describe the evaporation of the droplet: it assumes a uniform droplet temperature approach, where the process is mainly driven by fuel vapor concentration gradient at droplet surface. Here, the fuel vapor is assumed to be in equilibrium with the liquid phase at the local saturation conditions prescribed by droplet temperature. In such conditions, the evaporation rate is given by the following expression:

(14) $$\begin{array}{c} {\tilde{\dot{m}}}_{d}=-\pi d\rho DS_{h}B_{M}, \end{array}$$

where *d* is the droplet diameter, ρ and *D* are the density and mass diffusivity of the air-vapor mixture, and $B_{M}$ is the mass Spalding number [38]. The effect of forced convection over droplet surface is described by the Sherwood number $S_{h}$, which is dependent on Schmidt number and on particle Reynolds number. The transport properties of Jet A-1 fuel have been obtained by data reported in Reference [39].

### 4.4. Radiative Heat Transfer Modeling

The radiative heat transfer occurring in the combustor is computed by solving the Radiative Transfer Equation (RTE) with the Discrete Ordinate (DO) model [40]. In this approach, the RTE is solved in a polar coordinate system with a ray tracing strategy over a discrete number of $N_{\theta }\times N_{\phi }$ solid angles, which represent the beam directions. In order to make the solution of each RTE’s (one for each direction) coherent with the discretization of the fluid governing equations, a projection on Cartesian coordinate system is carried out. This discretization may induce some misalignments between face normal to fluid control volume and the solid angle, resulting in inaccurate computation of radiation intensity fluxes: a pixelation is adopted to fix this problem by dividing the solid angle in $N_{\theta_{p}}\times N_{\phi_{p}}$ pixels (4 × 4 angular discretization and 3 × 3 pixelation are used in this work). Concerning the spectral properties of the participating media, a Weighted Sum of Grey Gases approach was used. Further details about the adopted modeling are reported in Reference [41].

### 4.5. Computational Domains and Numerical Grids

As widely discussed in Section 2 and shown in Figure 1, the framework applied to this context requires solving convection, conduction, and radiation in three different instances of the software. The convective solver, the most critical from a numerical point of view, adopts a pressure-velocity coupling based on the SIMPLEC (Semi-Implicit Method for Pressure Linked Equations-Consistent) algorithm and a second-order discretization to improve the accuracy in both the advection and temporal contributions.

As a result of preliminary RANS simulations, a fluid time step of $3\cdot 10^{-6}$ s was set to properly solve the largest turbulence scales. This value can also ensure a CFL number around unity. Concerning the other time-dependent block, i.e., the conduction one, a time step of $1\cdot 10^{-3}$ s was imposed according to the estimated time scale of the phenomenon itself. The two aforementioned solvers were coupled with each other when a time-advancement of $3\cdot 10^{-5}$ s and $3\cdot 10^{-2}$ s were reached, respectively, for the fluid and solid domains, corresponding to an acceleration factor for the solid solver (i.e., the ratio between solid and fluid time-advancements) of 1000.

**Figure 9:**
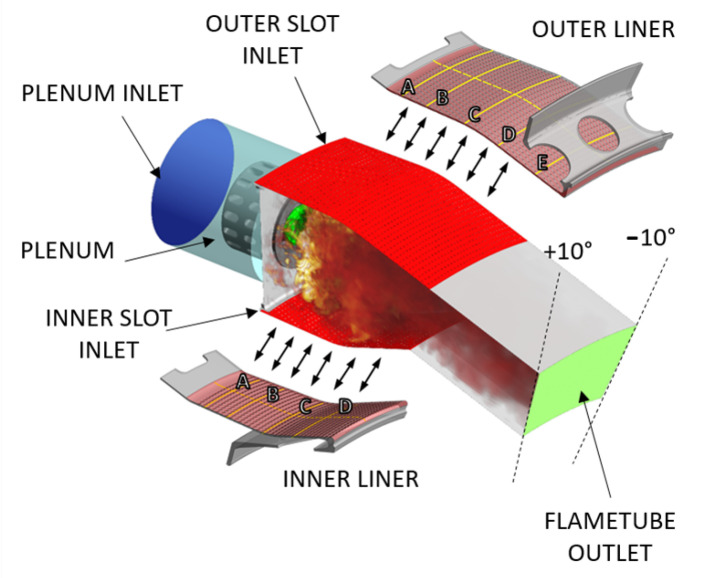
Computational domains and main boundary conditions. Uppercase A, B, C, D, E refer to the yellow lines where data are extracted for post-processing.

A sketch of the simulated computational domain, together with the applied boundary conditions, is depicted in Figure 9 and discussed here below.

#### 4.5.1. Domains

The potential of Scale-resolving applications is widely appreciated, where several phenomena are strongly coupled each other and strictly related to turbulence. In the present work, this condition can be found in the flametube that shows turbulence, combustion, and spray evolution. Moreover, the unsteadiness of the hot swirling flow has an indirect effect on the wall heat fluxes: this is a key point for the final target of obtaining a high-fidelity prediction of the liner thermal load. Hence, to speed-up the flow field simulation without an appreciable lack of accuracy, a domain including only the flametube and an upstream plenum was taken into account. An investigation of the whole system, including hot and cold sides, is illustrated in Reference [24] using a RANS approach, highlighting how the wall temperature is driven by the hot side. This domain choice has little or nothing effects on the radiative fluxes because the cold annulus does not see the flame and the contribution of radiation is negligible compared against the convective ones, as reported on the energy budgets in Reference [24].

The solid is solved using a different domain corresponding to the inner and outer liners. In this work, radiation through the wall thickness is not included because of the radiative properties of the liner, that can be considered opaque and, for this reason, absorbs and reflects the beams at the fluid-solid interface obstructing the transmission.

#### 4.5.2. Computational Grids

**Figure 10:**
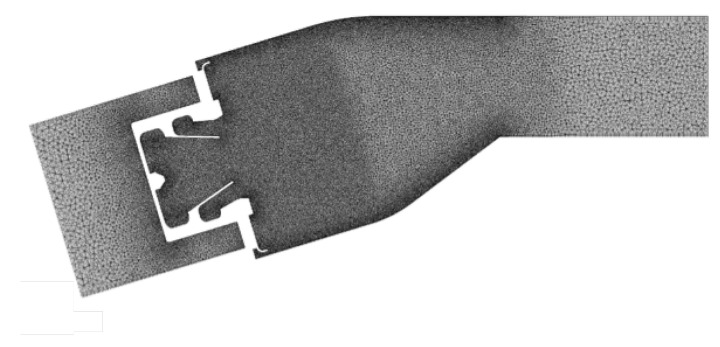
Computational grid exploited for the fluid domain.

Ad-hoc computational grids were generated in ANSYS Meshing for the three domains, with respect to the mesh requirements of each simulation. As the sensitivity on the mesh parameters is a time-consuming task in a multi-physics problem, being characterized by several meshes and unsteady simulations, the size of the grids were a trade-off between computational effort and accuracy. In particular, a computational grid built by 8.7 M tetrahedral elements was exploited for the flow field solution and is shown in Figure 10. Even if grid spacing has not a direct effect on the scale resolution in the SAS approach, its adequacy was deeply investigated in Reference [25] through a mesh sensitivity study, highlighting its potential in the prediction of the aerothermal field. In order to combine wall function methods [34] to the SAS model in the boundary layer zone, where the model behaves as a RANS *k*-ω SST model, a prismatic layer counting 3 elements was included, using a size that provided a $y^{+}$ adequate for this wall treatment.

Concerning solid and radiation grids, these were the same exploited in Reference [24]. In the second one, it was strongly coarsened starting from the mesh of the convective problem, obtaining a 4 M tet-only elements grid. The application both of a loose coupling and a mesh coarsening, can improve the computational efficiency of the coupled fluid-radiation problem of around 30–40%. The solid grid, instead, needs a huge refinement caused by the 2000 tiny holes of the effusion cooling system, resulting in 21.3 M tetrahedral elements.

### 4.6. Boundary Conditions

Following the experience gained in References [24,25], the definition of the boundary conditions was not a critical task. According to Reference [25], for the convective instance, mass flow inlet and pressure (static) outlet conditions were prescribed, as shown in Figure 9. Mass flow at the boundaries representing the coolant injection from the multi-perforation holes (i.e., the imprint of the holes) was determined through a flow split analysis made in preliminary RANS simulations, where the effusion liner was modeled through point mass sources using the SAFE (Source bAsed eFfusion modEl) methodology, presented in Reference [42] and applied also in Reference [43]. With the aim of making the pre-processing faster, in the present work, individual rows of effusion holes were grouped in a single patch where uniform conditions were applied. The inlet conditions in the upstream plenum, as well as the operating pressure and the fuel mass flow rate, were set according to the specific operating condition reported in Table 2. According to the opaque properties of the walls, the boundary conditions for the radiation domain consist of absorbing/emitting patches, not only for the walls but also concerning the inlets and outlets. Solid-gas coupling occurs on the red regions in Figure 9, corresponding to the liner hot sides of the two liners: for these interfaces, coupling BCs are required. At these locations, radiative and convective wall heat fluxes are calculated and translated according to Equations (1) and (2). As part of the U-THERM3D process, the resulting quantities are sent to the solid solver, which calculates the metal temperature and returns that distribution to the other solvers.

As the focus of the present work is on the estimation of the benefits of scale resolving approaches on the CHT problem applied to aeronautical gas turbine combustors, having neglected the solution of the cold side is justified. In the multi-perforation holes, instead, where the turbulence scale would be extremely small making the SAS solution infeasible, the U-THERM3D methodology is capable of exploiting a correlative approach to keep a good accuracy. As a result, heat transfer coefficients and reference temperatures coming from the THERM3D steady analysis on the full single sector geometry performed in Reference [24] were set on the aforementioned zones of the solid simulation. An analogous approach was adopted to impose the coolant temperature on the inlet patches representing the exit of the effusion holes. This assumption is justified by the small heat pick-up of coolant crossing the multi-perforation. The remaining walls were treated as smooth, no slip, and adiabatic.

### 4.7. Material Properties

Liner was treated as a metal alloy with temperature-dependent conductivity and heat capacity. A similar dependency by the temperature was set also in the fluid, where, in addition, the effect of a change in the gas composition caused by the combustion phenomenon was included trough a polynomial-dependency by progress variable and mixture fraction. Properties taken from [39] were used to characterize Jet A-1 fuel. The spectral radiation is approximated with a weighted sum of gray gases, while metal emissivity is set 0.8.

## 5. Results

In this section, the results obtained for the two test points will be presented and discussed, providing a comparison against the THERM3D results [24]. The results at the Approach condition are presented first, so as to provide a validation with the available experimental data, and then the Take-Off condition is considered to assess the impact of the increased aerothermal loads on metal temperature.

### 5.1. Approach

**Figure 11:**
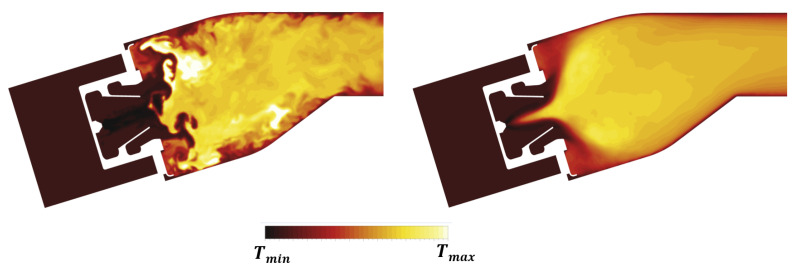
Contour maps of instantaneous (**left**) and time averaged (**right**) gas temperature at Approach condition.

The influence of the double swirler concept adopted in the PERM injection system was extensively discussed in previous works [25]. Its geometry determines the aerothermal field within the combustion chamber, determining a swirl-stabilized flame characterized by a large inner recirculation zone, as well as outer recirculation zones in the proximity of the corners between liners and dome. Given the operating conditions, most of the liquid fuel is injected through the pressure atomizer (see Table 2), generating droplets, thanks to the strong break-up process. The particles start their evaporation within the swirler due to the high velocity and temperature of the air, thus contributing to a periodic hot gases ingestion that takes place within the inner swirler. This behavior can be observed in the time-averaged temperature contours reported in Figure 11, whereas the particular snapshot chosen for the instantaneous temperature field seems temporarily unaffected by such phenomenon.

The core region of the combustion chamber seems not affected at Approach by the effects of the coupling with the radiative and thermal calculations if compared to the adiabatic simulation already reported in Reference [25]. The high velocity regions caused by the swirling flow generate significant shear stresses that promote the breakup of thin film generated by the fuel on the lip of the injection system. The turbulence of the velocity field then promotes the subsequent dispersion of the liquid droplets, as well as of the fuel, evaporated. Droplets and evaporated fuels trapped by large eddies into pockets are mixed and burnt, generating hot spots moving downstream. The interaction of these turbulent structures with the liners is inherently non-stationary and contributes to increasing convection and, ultimately, the metal temperature. The investigation of such a phenomenon in the context of on adiabatic simulations [24] highlighted the impact of turbulence modeling in RANS and SAS. In particular, differences were shown not only for the predicted mean adiabatic wall temperature but also for the wide range of its fluctuations especially in the upstream region of the liner. The redistribution of turbulent kinetic energy in the chamber plays a fundamental role in determining the wall heat transfer, in particular, in the outer recirculation regions, where the time averaged temperature returned by SAS is sensibly higher than the one obtained with RANS shown in Reference [24]. This effect can be undeniably ascribed to the SRSs that are able to solve a portion of turbulence spectrum and its effect of the turbulent diffusion.

**Figure 12:**
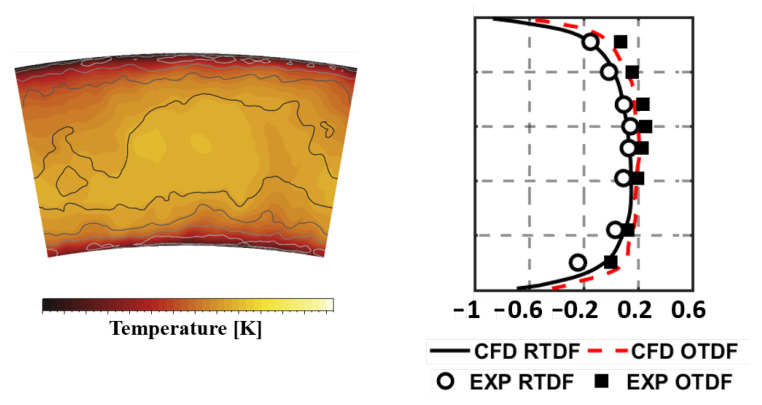
Time averaged temperature distributions at Approach on Plane 40 superimposed with iso-lines of temperature RMS normalized to the local averaged value.

The temperature distribution is also analyzed on the Plane 40 so as to provide more evidences about the reliability and robustness of the numerical methodology employed. Figure 12, on the left, reports the temperature field at Plane 40 in terms of both time averaged and instantaneous values. These are expressed as maps of the ratio of RMS value to the local mean temperature value. While the peak of mean temperature is located at midspan, the highest fluctuations are observed at lower and higher radii because of the presence of the film cooling. In order to obtain a more quantitative estimation and permit a direct comparison with experimental data, RTDF and OTDF in terms of 1-D profiles were obtained, according to Equations (15) and (16):

(15) $$RTDF\left(r\right)=\frac{\bar{T(r)}-T_{40}}{T_{40}-T_{30}},$$

(16) $$OTDF\left(r\right)=\frac{T{\left(r\right)}^{max}-T_{40}}{T_{40}-T_{30}}.$$

The results are reported in Figure 12, on the right, and show a classical parabolic distribution with relevant temperature gradients in the radial direction. This can be justified mainly by the lack of dilution jets, as well as by the presence of cool air layer in the near wall region injected through effusion. A fairly reasonable agreement is observed with experimental data, with only a slight overestimation of the RTDF.

**Figure 13:**
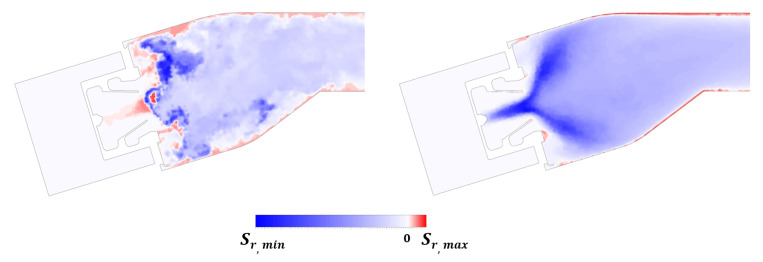
Contours of a time snapshot (**left**) and time averaged (**right**) energy source term due to radiation at Approach condition.

The fluctuating and averaged parts of the energy source term produced by radiation are reported in Figure 13, which represent the coupling data exchanged from the radiative to the convective simulation. Despite the contribution of species composition the absorption and emission properties of the participating media, gas temperature is certainly the most relevant quantity influencing the energy source term. The flame region has negative values (indicating that energy is lost from the radiative domain and transferred to the convective domain). As it is possible to observe by a comparison with Figure 11, regions with the highest gas temperature are interested by high negative energy sink, while low temperature regions, such as those interested by mixing with cooling air, are experiencing absorption of radiative energy.

**Figure 14:**
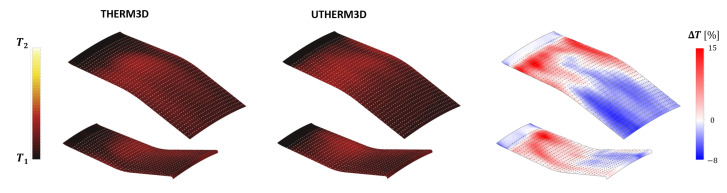
Contour maps of time averaged temperature on the gas side of the liners for the THERM3D (**left**) and U-THERM3D (**center**) simulations at Approach condition. The relative percentage difference between two data sets (normalized by the THERM3D values) is reported on the (**right**).

Moving the focus on the inner and outer liners, it is possible to demonstrate how solving part of turbulence spectrum affects the predicted convection and liner temperature distributions, when an unsteady coupling is adopted. Temperature maps on the gas side of the liners for THERM3D [24] (left) and U-THERM3D simulations (center) are reported in Figure 14: the percentage difference of predicted metal temperature by the two approaches are reported on the right (values are normalized with the THERM3D values). As expected, the U-THERM3D approach points out a wider high-temperature region and smoother gradients when compared to the steady state predictions. The turbulent flow, resulting from the interaction between swirling jet and combustor walls, strongly affects the film coverage of both plain slots and effusion cooling holes. In the steady THERM3D calculation, two cold spots appear on the liners, showing a limited distortion effect due to swirling flow, with film cooling maintaining a significant effectiveness. On the contrary, in the scale resolving calculation, such features vanish as a consequence of a wider aperture angle of the swirling jet: a temperature increase of about 15% is observed in the impact region over combustor walls with respect to THERM3D solution. In the regions just downstream each plain slot, similar temperature levels are observed, with the flow field being dominated by the corner recirculation zones. Both outer and inner liners show high temperature regions in the first half, with the peak located in the region where the swirling flow is impinging on the walls, while, in the successive half of the liners, the superposition of film cooling permits to lower metal temperatures.

**Figure 15:**
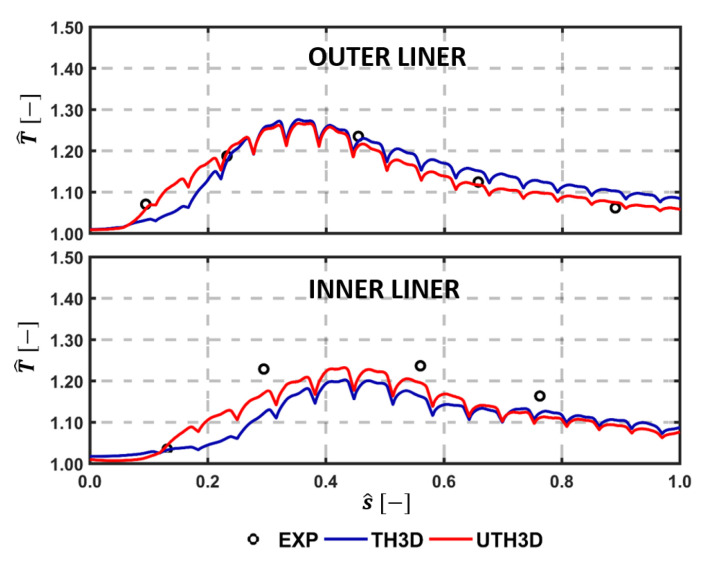
Temperature along centerline as per experiments, THERM3D, and U-THERM3D on the cold sides of the Inner (**top**) and Outer (**bottom**) liners at Approach condition.

**Figure 16:**
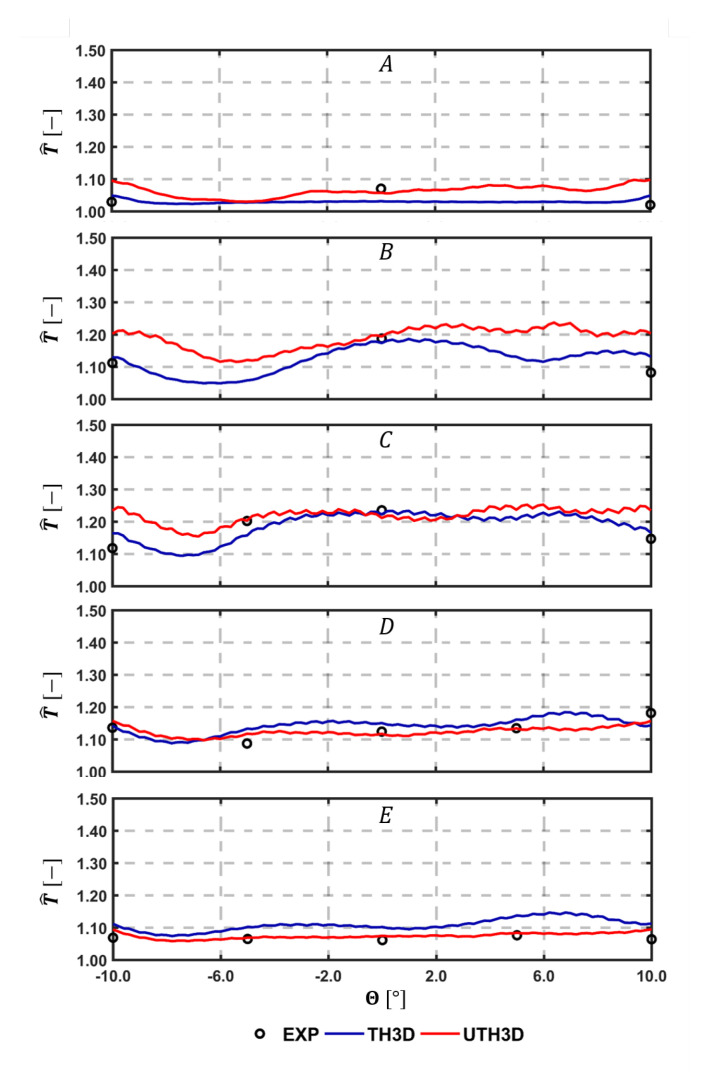
Tangential temperature distribution with comparison among test data, THERM3D, and U-THERM3D on the cold sides of the Outer Liner (locations are highlighted in Figure 9).

**Figure 17:**
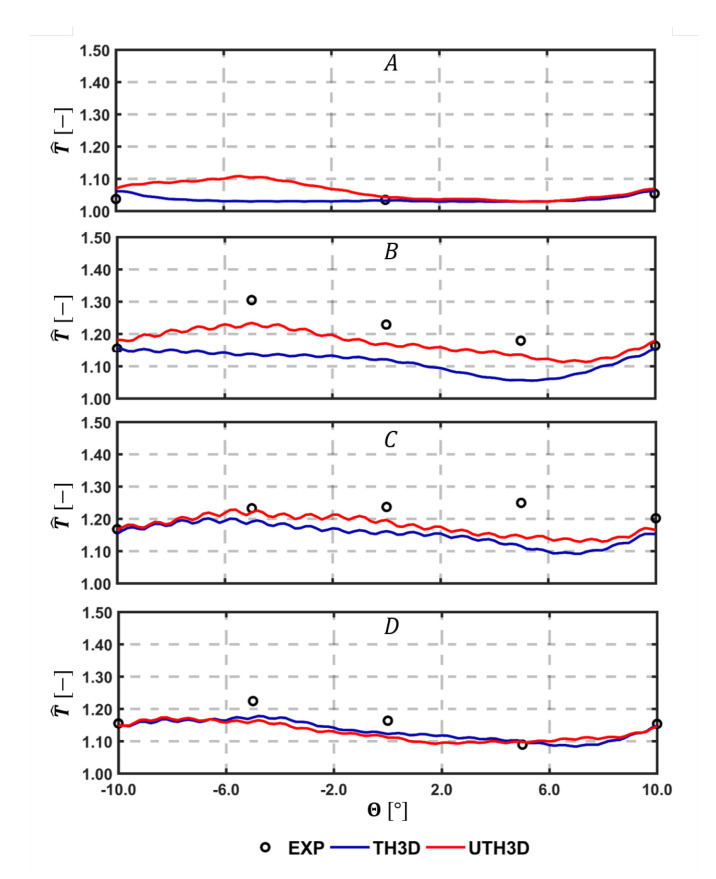
Tangential temperature distribution with comparison among test data, THERM3D, and U-THERM3D on the cold sides of the Inner Liner (locations are highlighted in Figure 9).

A quantitative comparison with experimental data is carried out by considering temperature measured on the cold side of the liners by thermocouples. Figure 15, Figure 16 and Figure 17 show the comparison among calculations and experiments: measuring points are extracted and the lines are pointed out in the scheme shown in Figure 9. The comparison points out the significant improvement obtained when switching to the SAS based multi-physics tool, especially on the outer liner. The U-THERM3D approach is capable of improving the prediction of more than 40% on the upstream side of the inner liner and to fully fit the experimental distribution on the outer liner compared against the steady THERM3D simulation.

The temperature distribution along the centerline for both inner and outer liners is reported in Figure 15 in terms of normalized curvilinear abscissa $\hat{s}$. The lower cooling effectiveness and higher heat loads predicted by the U-THERM3D approach in the first part of the liners are confirmed by the curves, as already discussed, as in Figure 14. Especially on the inner side, the numerical results are shifted towards measurements when exploiting the unsteady formulation of the coupling strategy. Even if the temperature of the outer liner was fairly well predicted by THERM3D, the present approach shows an additional improvement in the trend. For instance, a better agreement is obtained on the first measurement point, thanks to the predicted smoother temperature gradients.

Further similar comparisons can be carried out by looking at the measured data along tangential directions, as highlighted in Figure 9. Comparisons are shown in Figure 16 for the Outer Liner (lines A, B, C, D, E) and in Figure 17 for the Inner Liner (lines A, B, C, D). As already observed, the predicted smoother temperature gradients by U-THERM3D appreciably improve the accuracy in metal temperature prediction, with particular reference to locations B and C of the Inner Liner. Contrary to RANS, where the film protection is not affected by the swirling flow up to effusion row number 5, in the unsteady SAS calculation the coolant layer is destroyed by hot gases just upstream from effusion row number 2, bringing to high thermal loads at locations A, as confirmed by measured data. The predicted premature film disruption appears to not be fully confirmed at locations −10/10° of the Outer Liner, where metal temperature is overestimated. Such an effect could be ascribable to the boundary conditions applied to the effusion holes in terms of mass flow rate. In fact, a constant mass flow rate was prescribed for all rows, but, since the pressure is not uniform in the tangential direction, a uneven mass flow rate distribution would be more representative. Uneven distribution of pressure is expected, especially on the hot side of the liners due to the impinging swirling flow producing local stagnation effects. In particular, in the centerline region, the decreased of pressure drop across the liner would return a lower mass flow rate compared to the hypothesis of uniform injection. As a consequence, additional coolant would be injected from effusion jets in the midcup region (close to −10/10° locations), and the liner would be more protected in these regions. The effect of the uneven coolant distribution in tangential and axial directions on the heat load would deserve further investigations in the future.

### 5.2. Take-Off

**Figure 18:**
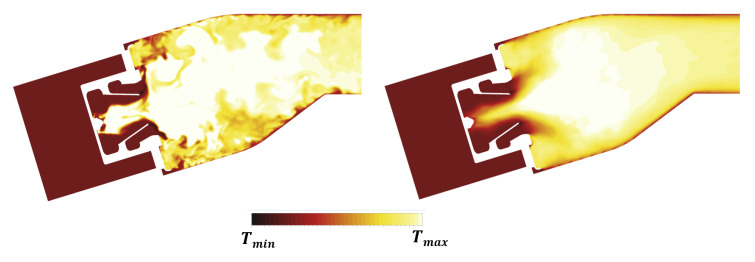
Contour maps of instantaneous (**left**) and time averaged (**right**) gas temperature at Take-Off condition.

Increasing values of FAR, P30, and T30 leads to higher heat release rates, gas temperatures, and, in the end, a more critical aerothermal environment for the liners. The hottest region of the flame shifts downstream towards the outlet, as it is possible to observe in Figure 18. Similarly to what pointed out at Approach, the flame tends to propagate backward inside air spray nozzle and reaches the proximity of the pilot fuel injector. This effect is visible both in instantaneous and time averaged gas temperature contours, even though monitoring the solution an oscillating behavior of the flame is still present within the swirler. Compared to the Approach conditions, the mean opening angle of the swirling flow appears reduced, as a consequence of the augmented flow rate. In addition, the greater fuel flow rate and turbulence intensity also brings the combustion process in the corners of the flametube.

**Figure 19:**
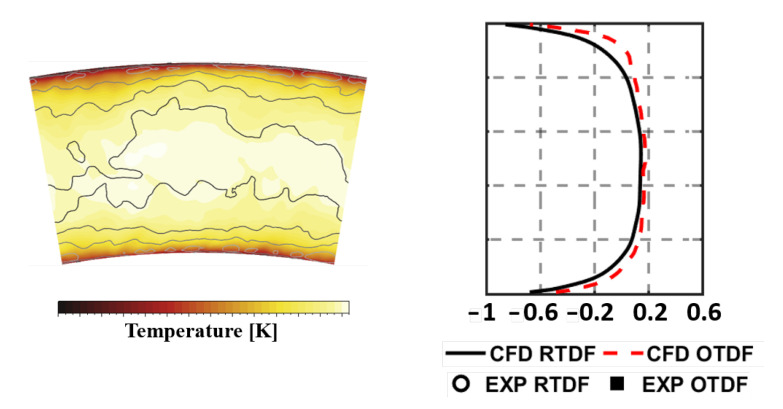
Time averaged temperature distributions at Take-Off on Plane 40 superimposed with iso-lines of temperature RMS normalized to the local averaged value.

Similarly to what was done for the Approach condition, the temperature fields analyzed on the Plane 40 are reported in Figure 19 in terms of contours of both mean and fluctuating values, as well as 1D profiles of RTDF and OTDF. Unfortunately, for these operating conditions, experimental data were not available. Despite that, it is possible to observe how the temperature is remarkably increased by the increased FAR and inlet temperature, while the 1D profiles are not affected in a significant way.

**Figure 20:**
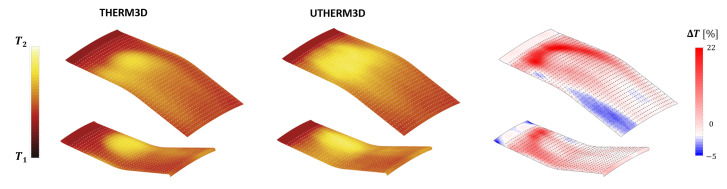
Contour maps of time averaged temperature on the gas side of the liners for the THERM3D (**left**) and U-THERM3D (**center**) simulations at Take-off condition. The relative percentage difference between two datasets (normalized by the THERM3D values) is reported on the (**right**).

The more severe conditions within the flametube cause a significant increase in the aerothermal loads on the liners compared to the Approach condition. This is clearly illustrated by the metal temperature distributions reported in Figure 20.

U-THERM3D predicts a considerably higher metal temperature compared to the values provided by the corresponding THERM3D simulation. A consistent increase in temperature can be observed almost everywhere. Peak values up to 20% in relative terms are located mainly in the upstream region of the liners, where the flame interacts with the walls. This is coherent with the different behavior predicted by RANS and SRSs in terms of opening angle of the swirling flow. Similarly to the Approach conditions, the hereafter remarks can be retained:

- the solution of part of the turbulence spectrum offered by SAS smooths the temperature gradients (and, thus, the thermal loads);
- with the resolved turbulent mixing more hot gases are transported into the outer recirculation zones, producing an increase in metal temperature of the liner in the proximity of the slot exit; and
- slot and film cooling flows are interacting more uniformly with the swirling flow in the spanwise direction, almost eliminating the cold coolant streaks reproduced by THERM3D.

Focusing on the relative temperature difference between unsteady and steady simulations, the downstream portion of the Outer Liner shows an opposite trend compared to the previous condition. At Approach, this region appears heated by the hot gases which, however, have exchanged a significant amount of heat with the primary zone of the liner and, for this reason, have lost part of their temperature. The SAS calculation further increases such heat transfer in the primary zone, thus resulting in even colder gases and ultimately fairly lower metal temperature. On the contrary, at Take-Off the flame is shifted more downstream and has, indeed, more heating potential, with hot radiating gas pockets convected to the last portion of the liner.

**Figure 21:**
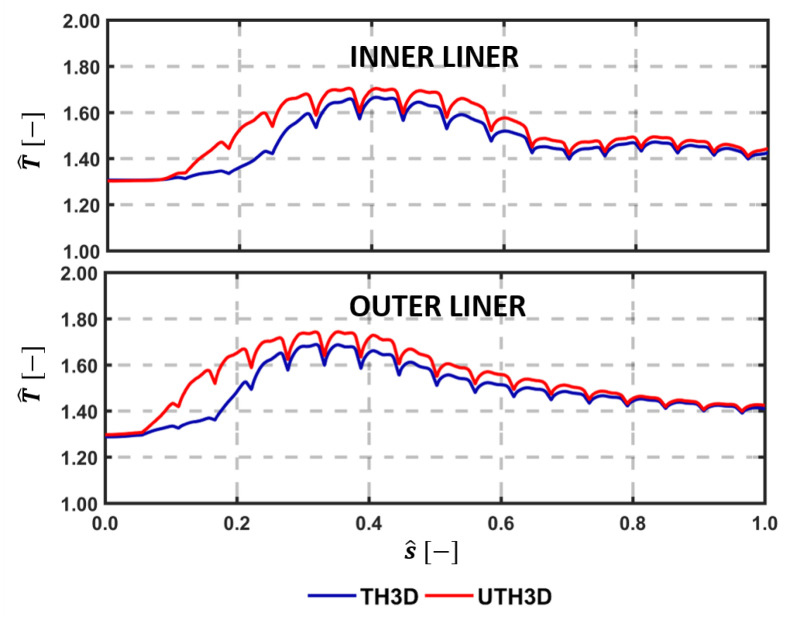
Temperature along centerline as per experiments, THERM3D, and U-THERM3D on the cold sides of the Inner (**top**) and Outer (**bottom**) liners at Take-Off condition.

Similar to Figure 15, in Figure 21 is reported the metal temperature distribution on the centerline of the flametube. The temperature peak identifies the swirling flow-wall interaction phenomenon, located approximately at 40% of the curvilinear coordinate for both liners and operating conditions. However, the temperature rise associated to the interaction between liner and flame is anticipated when considering the U-THERM3D calculation. This confirms, again, the great impact of a scale-resolving prediction on the metal temperature, especially in the outer recirculation zones. Another aspect worth to be remarked is the second small peak region appearing at 80% of the inner liner length, that is visible with both computational approaches. This feature seems caused by the radiative heat transfer, which is positively affected by an increased shape factor at Take-Off due to the longer flame. The view factor between the flame and the second half of the Inner Liner is not favorable because of the liner shaping related to its inclination, causing a local increase in the radiative heat flux.

### 5.3. Heat Load Analysis

**Figure 22:**
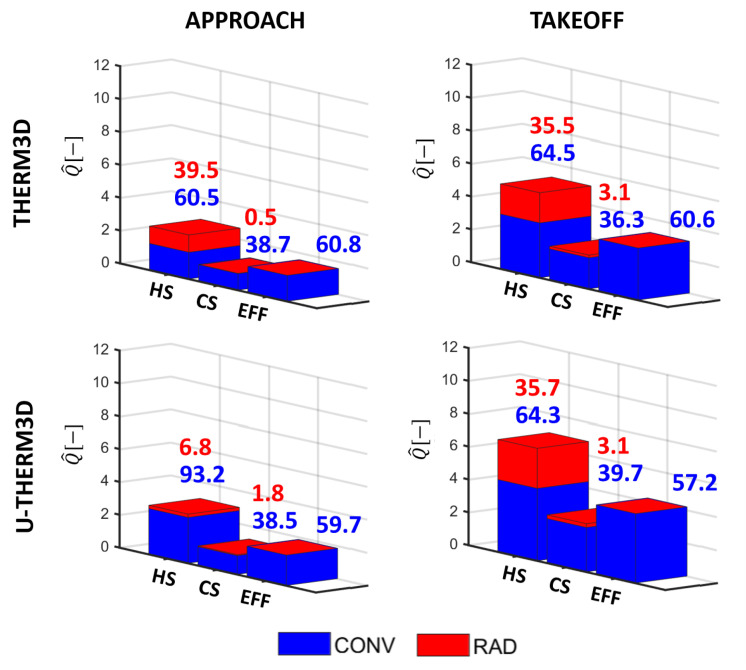
Comparison between THERM3D and U-THERM3D of the normalized total heat loads for the Inner Liner at Approach and Take-Off conditions. Values above the bars are the relative contribution of convection and radiation to the heating and cooling of the liner.

**Figure 23:**
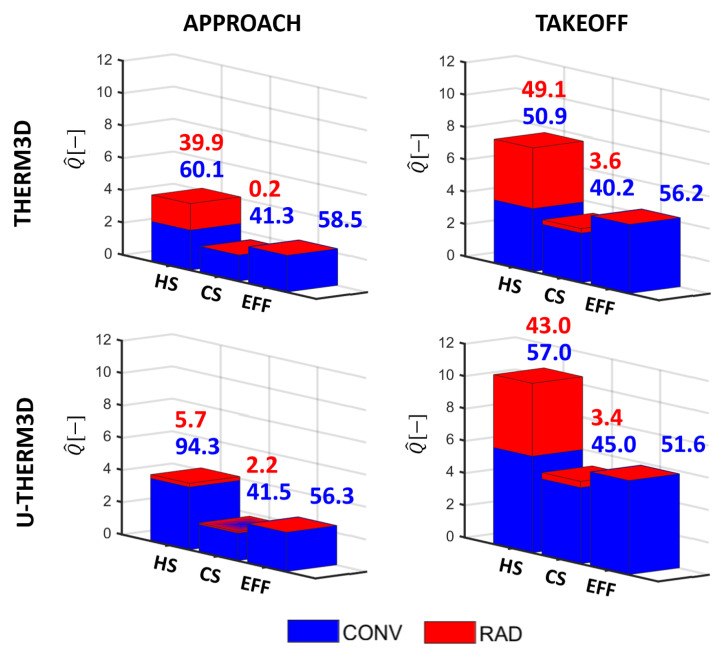
Comparison between THERM3D and U-THERM3D of the normalized total heat loads for the Outer Liner at Approach and Take-Off conditions. Values above the bars are the relative contribution of convection and radiation to the heating and cooling of the liner.

The different heat transfer modes (i.e., convection and radiation) have different contributions not only when different operating conditions are considered but also on the different regions of the liner (i.e., hot side, cold side, and within the perforation). Obtaining a deeper insight about the role played by the two mechanisms can help the designers in better understanding the metal temperature trends. At this purpose, the energy budget for Inner Liner and Outer Liner were calculated and are reported in Figure 22 and Figure 23. The total heat load was normalized by a reference value and partitioned into a convective (blue) and radiative (red) contribution. Then, a further split was done to quantify the hot side (HS), the cold side (CS), and the effusion holes (EFF). It is worth pointing out that the radiative contribution in the perforation is neglected in the current modeling. The values calculated are then compared against the results previously obtained with THERM3D for the same operating conditions. Above each bar, numbers are reported to provide, in a quantitative way, the relative contribution of convective and radiative heat transfer on the heating (HS) and cooling (CS+EFF) of the liners. The heat load is directly proportional to the temperature trends shown above, with a significant increase switching from Approach to Take-Off condition, independently by the coupling strategy adopted. Despite that, the coupling strategy based on unsteady simulations seems to modify the relative weight of the heat transfer modes compared to the steady framework. At the Approach condition, the radiative contribution is reduced in the U-THERM3D simulation by the lower gas temperature. In addition, the increased convection caused by a more reliable prediction of the interaction between swirling flow and liner increases the metal temperature and makes radiation almost negligible. At Take-Off, on the contrary, the SAS computation returned a more extended flame that leads the radiative heat load to grow, if compared to both the Approach condition and the THERM3D results at the same condition.

Overall, the heat load is more than doubled as a consequence of the different operating condition, even closer to a three-fold increase on the Outer Liner. As explained, the different distribution of radiative heat flux between the two liners can be addressed by the shaping of the annular geometry, that generates a higher viewing factor from the Inner surface and the flame to the Outer surface, while the Outer surface interacts with the Inner part with a lower viewing factor. As a consequence, the Inner Liner fully irradiates to the opposite surface that, contrarily, partly self irradiates. Non-uniformities in liner-to-liner radiation are even more significant at Take-Off, when solid temperatures are higher. This outcome is also confirmed by the relative contributions of radiation and convection which are, respectively, 35%/65% for the Inner Liner and 45%/55% for the Outer Liner.

Figure 22 and Figure 23 show quite evidently that the cooling effect is largely to be ascribed to the internal heat-pick within the effusion perforation, contributing to liner cooling for the 56% on average. The absolute value is barely affected at Approach by the coupling procedure adopted, but the increase is more evident at Take-Off, for which U-THERM3D predicts a higher metal temperature.

## 6. Conclusions

Conjugate heat transfer is a challenging problem, made even more complex in gas turbine combustors by multi-physics interactions between fluid dynamics phenomena, heat conduction and radiation. Experimental campaigns on real aeroengine burners cannot provide a deep insight into the involved phenomena. The more and more widespread use of massively parallel resources is pushing CFD to support the detailed design of modern combustors. Among all the requirements involved in their design, the management of metal temperature is one of the most relevant.

This article reports the main outcomes of a modeling activity finalized at investigating the thermal loads on lean burn combustor cooled by multiperforated liners. A desynchronized loosely-coupled approach, called U-THERM3D, was developed to reduce the computational effort of the computational intensive multi-physics problem. The tool was applied to a full-annular test case from a EU-funded research program. The work represents the continuation of previous preliminary tasks focused on steady and unsteady contexts. The Approach and Take-Off operating conditions of the aeroengine were considered. The results of the present methodology were compared with the predictions obtained with the steady multi-physics tool THERM3D [24], as well as measurement data at the Approach condition.

Despite the greater computational cost (roughly one order of magnitude higher than the steady version), the new tool returns an improvement of the accuracy in the prediction of metal temperature, as confirmed by the comparison with experimental data at Approach condition, with the overall prediction of higher metal temperatures and reduced associated gradients. As a consequence to a more accurate and physical prediction of the unsteady interaction between the swirling annular jet and combustor liners, common to both investigated operating conditions, higher temperature levels are computed in the first half of the liner with the SAS approach. As a matter of fact, the hot gases entrainment in the corner recirculation regions, the related increase in the wall heat flux, and the unsteady wash-out of film cooling is better reproduced by the Scale-Resolving Simulation approach. A heat load analysis reveals opposite trends for radiative heat transfer in the two operating conditions when the unsteady coupling is considered in place of the steady one. At the Approach condition, maximum gas temperature is not altered with respect to the THERM3D calculation and, contemporary to the higher liner temperature, it justifies a reduction of radiation. On the contrary, at Take-Off the radiative transfer slightly increases due to a larger flame volume in the reaction zone. Not far from what observed with the steady THERM3D solution, the ratio between convection and radiation is roughly 65%/35% and 55%/45% for the Inner and Outer liners, respectively. The greater contribution of radiation, for the latter case, can be motivated by the particular shaping of the annular combustor, which involves higher self view factors to the outer concave line. Therefore, the use of Scale Resolving simulations can reveal different changes in the relative contributions of heat transfer routes compared to a steady approach. The improved prediction of liner temperature observed with U-THERM3D confirms the potential of this approach for a affordable high-fidelity aerothermal design of aeroengine combustion chambers. Considering the tool as a framework for exploitation of models in a computationally-affordable way, the accuracy of the investigation can further benefit from the improvement of the adopted models (such as switching from SAS to LES, or the use of more advanced combustion models or detailed radiative spectral models). The authors feel that further research efforts should be focused on these tasks.

## Figures and Tables

**Table 1:** Solid properties for the backward-facing step problem.

Density [kg/m${}^{3}$]	Specific Heat [kJ/(kgK)]	Thermal Conductivity [W/(mK)]
100	50	5

**Table 2:** Details of the analyzed test points. Underlined values represent conditions adopted in the CFD simulations.

Test Point	P30	T30	FAR	P/T	Active
	bar	K	‰	%	Injectors
Approach (ICAO 30)	13.5	655	17.2	70	18
Take-Off (ICAO 100)	19.0	840	28.3	10	18

## Data Availability

Not applicable.

## Abbreviations

The following abbreviations are used in this manuscript:

$B_{M}$	Mass Spalding number [−]
*c*	Progress variable [−]
*d*	Droplet diameter [m]
*D*	Mass diffusivity [m${}^{2}$ s${}^{-1}$]
*L*	Length scale [m]
*P*	Pressure [Pa]
P/T	Pilot-to-total fuel split [%]
$q^{\prime \prime }$	Heat flux [W m${}^{-2}$]
$S_{r}$	Source of radiative energy [W m${}^{-3}$]
$S_{h}$	Sherwood number [−]
*T*	Temperature [K]
We	Weber number [−]
*s*	Curvilinear abscissa [m]
*Y*	Species mass fraction [−]
*Z*	Mixture fraction [−]
ACARE	Advisory Council for Aeronautics Research in Europe
BC	Boundary Condition
CAEP	Committee on Aviation Environmental Protection
CFD	Computational Fluid Dynamics
CHT	Conjugate Heat Transfer
CIAM	Central Institute of Aviation Motors
DES	Detached Eddy Simulation
DO	Discrete Ordinate
FAR	Fuel Air Ratio
FGM	Flamelet Generated Manifold
ICAO	International Civil Aviation Organization
LES	Large Eddy Simulation
NSE	Navier Stokes Equation
NEWAC	NEW Aero engine core Concepts
OPR	Overall Pressure Ratio
PDF	Probability Density Function
PERM	Partial Evaporation and Rapid Mixing
RANS	Reynolds Averaged Navier Stokes
RTE	Radiative Transfer Equation
SAFE	Source bAsed eFfusion modEl
SAS	Scale Adaptive Simulation
SRS	Scale-Resolving Simulation
TIT	Turbine Inlet Temperature
UDF	User Defined Function
α	Inclination angle (streamwise direction) ${[}^{\circ }]$
ω	Turbulence frequency [s${}^{-1}$]
Θ	Spanwise angle ${[}^{\circ }]$
ψ	Generic variable [−]
ρ	Density [kg m${}^{-3}$]
40	Referred to Plane 40 (combustor exit)
*c*	Progress variable
eq	Equilibrium
g	Gas phase
rad	Radiation
vK	von Karman
w	Wall
